# The effects of long- or short-term increased feed allowance prior to first service on litter size in gilts

**DOI:** 10.1093/tas/txab005

**Published:** 2021-01-15

**Authors:** Thomas S Bruun, Julie K Bache, Charlotte Amdi

**Affiliations:** 1 SEGES Danish Pig Research Centre, Copenhagen, Denmark; 2 Department of Veterinary and Animal Sciences, Faculty of Health and Medical Sciences, University of Copenhagen, Frederiksberg, Denmark

**Keywords:** altrenogest, feeding level, flushing, gilts, reproduction, total born

## Abstract

Replacing stock is costly in any pig production. In addition, it takes time for young animals to reach the same level of productivity as more mature animals. Therefore, the aim of this study was to investigate the effects of long- or short-term increased feed allowance (covering the luteal and follicular phases) prior to service in the second estrus on first parity performance. In order to achieve this, altrenogest was used to synchronize the gilts cycle to allow a precise feeding strategy, and only gilts inseminated 0–10 d after altrenogest withdrawal were included in the study. Altrenogest was given at days 0–18 to control the luteal phase and, therefore, treatments covered different feeding strategies in either or both the luteal phase (days 0–18) and follicular phase (days 18–25). High feed allowance (H) was induced using 0.97 kg more feed per day compared to the low feed allowance (L) given 2.33 kg/d. Four feeding strategies, low–low (LL), high–high (HH), high–low (HL), and low–high (LH), were included. Once gilts had been inseminated, feed allowance was reduced to 2.23 kg/d to prevent the loss of embryos in early gestation. A tendency was observed between feeding strategy and backfat thickness before altrenogest treatment, showing that total born piglets were positively correlated to backfat in the LL and LH (no increased feed allowance or short-term increased feed allowance), treatments (*P* = 0.076), compared to when gilts had longer periods with high feed allowance (HH and HL). High feed allowance in the follicular phase (LH) tended to increase the number of total born piglets compared to the other groups (*P* = 0.069) when applied in the follicular phase of the second standing estrus after the gilts were given altrenogest. This would be equivalent to the last 5–7 d of a 21-d cycle in gilts. The three other feeding strategies, comprising either the luteal and follicular phases (HH) or the luteal phase (HL) or none (LL), did not increase litter size. The weight of the gilt when entering the insemination section also had an effect on total born piglets (*P* < 0.001) with an increase in litter size with increased weight of the sow, but no differences between treatments. In conclusion, the weight of the gilt had an influence on the total litter size and gilts with low backfat tended to respond more positively to a longer period with high feed allowance than fatter gilts.

## INTRODUCTION

The replacement of sows has a great economic impact at the farm level (Rodríguez et al., 2011). In addition, it takes time for young animals to reach the same level of productivity as more mature animals. For example, in sows, the productivity is one or two piglets less in the first litter (Le Cozler et al., 1998; Bruun et al., 2016) and the average litter weight is lower at weaning (Strathe et al., 2017) compared to subsequent litters. In order to improve the productivity of the first litter, it is recommended to inseminate the gilt in the second estrus as this increases the litter size by 1.1–1.4 piglets per litter compared to inseminating in the first estrus (Beltranena et al., 1991a, 1991b). This is due to a higher ovulation rate after the gilts’ first estrus cycle (Beltranena et al., 1991b). Moreover, producing replacement gilts is feed costly, takes up space, and a weaned sow is, therefore, more profitable to have in the production system compared to a gilt that has been fed throughout the growing period till maturation.

Generally, there is a positive effect of increasing the feed allowance just before insemination, often referred to as flushing (Close and Cole, 2000), which ensures a positive energy balance. Langendijk (2015) suggested that not only does flushing increase the amount of ovulated follicles but there is also a carryover effect of flushing on the release of progesterone after insemination, which has a positive effect on embryo survival. This is due to more evenly developed follicles and more follicles could potentially increase the release of progesterone (Langendijk, 2015). Some studies have shown that increasing the feed allowance in the luteal phase could also influence the maturation of the follicles that are released in the following cycle (Hazeleger et al., 2005; Chen et al., 2012). This is due to the fact that, in the last part of the luteal phase, there is a prefollicular phase that starts 4–6 d before the follicular phase where the growth of the follicles that are ovulated in the upcoming estrus slowly starts to grow. This can potentially have a large influence on both the quality and amount of follicles that grow in the follicular phase (Soede et al., 2011).

Current Danish recommendations state that feed allowance should be increased from approximately 2.9 kg to approximately 3.5 kg/d 7–14 d prior to insemination. This broad interval covers both the luteal phase that lasts approximately 15 d of the gilts cycle [where corpus luteum (CL) forms after ovulation] and the follicular phase [where new follicles start developing after CL has started to break down], which covers the last 5–7 d of a 21-d cycle (Soede et al., 2011). Providing a high feed allowance for gilts for a longer time period is not beneficial as this is both economically expensive and increases the risk of heavy gilts and subsequent locomotor problems (Amaral Filha et al., 2009; Kummer et al., 2009; Patterson et al., 2010).

Therefore, the aim of this study was to investigate the effects of long- or short-term increased feed allowance (covering the luteal and follicular phases) prior to service in the second estrus on first parity performance. Altrenogest was used to synchronize the gilts cycle in order to allow a precise feeding strategy for individually housed gilts, and only gilts inseminated 0–10 d after altrenogest withdrawal were included in the study.

## MATERIAL AND METHODS

All gilts originated from a commercial production facility. The health and welfare of all animals were monitored daily throughout the study by the farm staff according to the site’s standard operating protocols and veterinary recommendations and all usual practices for treatments, vaccinations, and management were followed. In addition, 24-h farrowing surveillance was performed on the farm. The farm complex has a state document on the well-being and welfare of the herd—on the assignment of the highest degree of biological protection—compartment no. 4. Additionally, the farm was under the supervision of a state veterinarian who monitors animal health. All procedures were carried out in compliance with federal orders and did not require separate approval by state structures since this was not a trial related to introducing infections or violence against animals. The study was carried out in the format of a field test; state registration of the study was not prescribed by law and was not necessary for the approval of the results of the study (orders of the Ministry of Agriculture of Russia 114 and 258).

### Herd and Animals

The study was conducted from May 2017 to December 2017 in a 6,000 sow commercial Russian piggery located in the Kaliningrad Region that produced their own (*n* = 1026) Danish Landrace × Danish Yorkshire gilts (DanBred, Herlev, Denmark) with a mean litter size of 15.5 (±3.1) total born. Halfway through the study, the piggery changed to receiving gilts (*n* = 1071) from another site but with the same breed (Danish Landrace × Danish Yorkshire gilts; DanBred, Herlev, Denmark) with mean litter size 16.7 (±3.0) total born. Management and dietary treatments were the same before and after the change of herd origin of the gilts.

### Pretreatment of Gilts

In the gilt-growing period from approximately 80 kg, the gilts were housed in pens (fully slated 5.0 × 2.95 m) with 16 gilts per pen. The stable was ventilated using negative pressure ventilation through wall inlets. Gilt feed was provided ad libitum, all feed was produced on farm, and the mineral premix was purchased from Vilomix (Vilomix, Mørke, Denmark). The main ingredients were wheat, barley, wheat bran, and soybean meal and contained 6.1 standardized ileal digestible (SID) lysine/kg, 110 g SID CP/kg, and 9.0 MJ NE/kg. Further details are presented in Table 1.

**Table 1.** Ingredients and nutrient composition of the three diets used for gilts in the rearing unit from 80 kg and in the breeding unit until day 28 of gestation, for gestating sows throughout gestation (days 29–112), and for sows from entering the farrowing unit at day 113 of gestation until weaning

	Diet		
	Gilts	Gestation	Lactation
Ingredient, g/kg “as-fed”			
Barley	150	274	210
Wheat	427	478	472
Wheat bran	150	3.1	–
Oat	–	47.7	21.4
Rye	100	–	–
Sugar beet pulp	50.0	70.4	20.1
Alfaalfa pellets	20.0	20.0	20.0
Soy bean meal	33.0	–	162
Sunflower meal	25.0	67.9	10.4
Soy oil	10.0	8.3	35.1
L-Lys	3.34	3,39	4.37
DL-Met	0.04	0.04	1.15
L-Thr	0.79	0.80	1.80
L-Trp	0.05	0.05	–
Monocalcium phosphate	8.8	7.6	13.6
Limestone	9.7	9.5	12.5
Salt	4.7	4.6	5.4
Vitamin and mineral premix	7.58^*a*^	7.69^*b*^	10.18^*c*^
Composition (calculated)			
DM, %	87.4	88.0	88.4
CP, %	110.2	104.0	147.1
SID^*d*^ CP, g/kg	6.1	5.5	9.6
SID^*d*^ Lys, g/kg	2.1	1.9	3.0
SID^*d*^ Met, g/kg	4.3	4.0	5.6
SID^*d*^ Met + Cys, g/kg	4.2	3.8	6.2
SID^*d*^ Thr, g/kg	1.4	1.3	1.8
SID^*d*^ Val, g/kg	4.8	4.5	6.5
Energy, FUsow/kg^*e*^	1.03	1.0	1.14
Energy, MJ NE/kg^*f*^	9.0	9.0	9.8
Composition (analyzed)^*g*^			
DM, %	85.7 (87.4)	n.a.	n.a.
CP, %	14.3 (13.8)	n.a.	n.a.
Lys, g/kg	7.6 (7.3)	n.a.	n.a.
Met, g/kg	2.4 (2.5)	n.a.	n.a.
Thr, g/kg	5.3 (5.3)	n.a.	n.a.
Val, g/kg	6.1 (6.2)	n.a.	n.a.
Ca, g/kg	8.4 (7.2)	n.a.	n.a.
P, g/kg	6.5 (6.1)	n.a.	n.a.

n.a., not analyzed.

^*a*^Provided per kilogram of the diet: 10,490 IU vitamin A; 2,000 IU 25-hydroxy vitamin D3 (HyD, DSM Nutritional Products, Basel, Switzerland); 104.9 mg DL-alfatocoferol, 4.2 mg vitamin K3; 2.1 mg vitamin B1; 5.2 mg vitamin B2; 3.1 mg vitamin B6; 0.03 mg vitamin B12; 15.7 mg D-pantothenic acid; 21.0 mg niacin; 1.6 mg folic acid; 83.9 mg iron (FeSO_4_); 17.8 mg copper (CuSO_4_); 104.9 mg zinc (ZnO); 47.2 mg manganese (MnO); 1.0 mg iodine (Ca(IO_3_)_2_); 0.31 mg selenium (Na_2_SeO_3_); 264.4 mg choline chloride.

^*b*^Provided per kilogram of the diet: 10,700 IU vitamin A; 2,000 IU 25-hydroxy vitamin D3 (HyD, DSM Nutritional Products, Basel, Switzerland); 106.7 mg DL-alfatocoferol, 4.3 mg vitamin K3; 2.1 mg vitamin B1; 5.3 mg vitamin B2; 3.2 mg vitamin B6; 0.03 mg vitamin B12; 16.0 mg D-pantothenic acid; 21.3 mg niacin; 1.6 mg folic acid; 85.4 mg iron (FeSO_4_); 18.1 mg copper (CuSO_4_); 106,7 mg zinc (ZnO); 48.0 mg manganese (MnO); 1.0 mg iodine (Ca(IO_3_)_2_); 0.32 mg selenium (Na_2_SeO_3_); 268.9 mg choline chloride.

^*c*^Provided per kilogram of the diet: 9,200 IU vitamin A; 2,000 IU 25-hydroxy vitamin D3 (HyD, DSM Nutritional Products, Basel, Switzerland); 190.2 mg DL-alfatocoferol, 4.6 mg vitamin K3; 2.3 mg vitamin; B1; 5.8 mg vitamin B2; 3.5 mg vitamin B6; 0.03 mg vitamin B12; 17.3 mg D-pantothenic acid; 23.1 mg niacin; 1.7 mg folic acid; 86.6 mg iron (FeSO_4_) + 86.6 mg iron (C_4_H_2_FeO_4_); 17.3 mg copper (CuSO_4_); 46.2 mg manganese (MnO); 1.2 mg iodine (Ca(IO_3_)_2_); 0.35 mg selenium (Na_2_SeO_3_) + 0.12 mg selenium (selenium yeast); 288,7 mg choline chloride.

^*d*^Planned SID values were calculated based on individual SID coefficients of feed ingredients used in the diet according to Pedersen and Boisen (2002).

^*e*^FUsow refers to Danish feed units for sows calculated in the Danish feed evaluation system (potential physiological energy system) closely related to the NE system (Patience, 2012).

^*f*^Energy in NE/kg was calculated from table values using EVA Pig.

^*g*^Values in brackets are expected values.

### Randomization of Gilts

In the gilt-rearing unit, gilts were daily selected to the groups by registering visual signs of pre-estrus or estrus [pink and swollen vulva or showing standing response as described by Soede and Kemp (1997)]. No boar contact was provided in the gilt-rearing unit, and hence, gilts that showed signs of pre-estrus or estrus were then consecutively allocated to one of the four treatments. The allocation sequence of the four treatments was varied from pen to pen to ensure that gilts entering the breeding unit at 216 ± 4 d of age had the same mean age and average day in their cycle across the four treatments.

### Preinsemination Housing, Management, and Feeding Strategies

Once a week, gilts with observed signs of either pre-estrus or estrus were moved from the gilt rearing unit to the breeding unit and were weighed (3- × 2-m weighing platform, Phiztech, Russia) and backfat scanned at the last rib and 65 mm from the backbone of the gilt (P2 site) using an ultrasound scanner (LeanMeater, Renco Corporation, Minneapolis, MN). In the breeding unit, the gilts were individually housed in pens (230 × 55 cm) with ad lib access to water via a drinking nipple. The gilt breeding unit was ventilated using negative pressure ventilation through wall inlets. From entering the breeding unit and until the four distinct feeding strategies were initiated 4 d later, all gilts were fed a fixed amount of 2.34 kg/d of feed irrespective of body weight. Approximately 2 d after the gilts were moved to the breeding unit, they were given an oral administration of apple juice (5 mL) with a syringe to adapt to the subsequent altrenogest (orally active synthetic progestogen; allyltrenbolone) treatment. All gilts were thereafter given an oral dose of altrenogest (5 mL Altresyn, 4 mg altrenogest/mL, Ceva Animal Health, Libourne, France) for 18 d. Gilts with less than 9 mm of backfat when entering the breeding unit were not included in the study. Also, gilts with locomotive problems, abnormal discharge, or coughing in the period housed in the breeding unit were also excluded.

### Dietary Strategies

The study was conducted as a block design with four dietary strategies prior to the first service in the second detected estrus. The first day of altrenogest treatment was considered as day 0 of the trial, and the dietary strategies were applied from day 0 to day 18 (considered as the luteal phase due to altrenogest treatment) and from day 19 and until insemination (considered as the follicular phase). The four strategies differed in providing either a low energy level (L; 2.33 kg/d) or high energy level (H; 3.30 kg/d) in the luteal and follicular phases, respectively. The combinations used were LL, HH, HL, and LH (Table 2).

**Table 2.** Feeding strategy (kilograms per gilt per day) from entering the breeding herd and until farrowing for all treatment groups (low–low energy levels, LL; high–high energy levels, HH; high–low energy levels, H; and low–high energy levels, LH)

	Group			
Time period	LL	HH	HL	LH
Breeding unit				
From entry until start of altrenogest treatment (day 0)^*a*^	2.33	2.33	2.33	2.33
Days 0–18	2.33	3.30	3.30	2.33
Day 19 until insemination	2.33	3.30	2.33	3.30
Total days with high feeding level, days	0	23–25^*d*^	18	5–7^*d*^
Gestation days 0–28	2.23	2.23	2.23	2.23
Gestation unit				
Gestation days 29–95^*b*^	1.8	1.8	1.8	1.8
Gestation days 96–112	3.5	3.5	3.5	3.5
Farrowing unit				
Gestation day 113 and until farrowing^*c*^	3.07	3.07	3.07	3.07

^*a*^Day 0 is the first day that the gilts received altrenogest treatment.

^*b*^In this time period, the gilts were fed according to backfat thickness, and gilts deemed less than average were given 2.3 kg/d, while gilts above average in body condition were given 1.8 kg/d.

^*c*^After entry into the farrowing unit, the gilts received lactation feed.

^*d*^The total days with high feeding level was dependent on the number of days from end of altrenogest treatment until insemination.

### Insemination Strategy

Altrenogest treatment gilts were checked for signs of estrus twice daily. This included fenceline stimulation with three boars, one after another, and for each individual boar, contact gilts were checked for standing response by the personnel until this was confirmed or declined. When standing response was confirmed, the gilts were inseminated with 24-h intervals for as long as they were showing standing response. Gilts were mated with DanBred Duroc semen (2.5 × 10^9^ semen per dose in a volume of 95 mL) of internally bred boars.

### Postinsemination Management and Feeding Strategies

After the last insemination, the gilts remained in the breeding unit and the feed allowance was reduced to 2.23 kg/d for all gilts the next 28 d as this was the normal practice in the herd after insemination. The gilts were fed with pelleted feed twice a day (0800 and 1300 h) for the first 28 d after insemination. After day 28 the gilts were moved to the gestation unit and were fed according to their backfat depth with gestation feed (Table 1) twice a day (0800 and 1300 h). Gilts with a body condition (backfat thickness) below the average were given 2.3 kg/d, while gilts above average in body condition were given 1.8 kg/d.

In the gestation section, there was an individual feeder/eating space per gilt and an activity area with full slatted floor behind the eating space. Approximately 5 d before the expected farrowing, the gilts were moved to the farrowing section where they were given a lactation feed (Table 1) three times a day (0815, 1130, and 1530 h).

### Recordings

After the gilts were moved to the gilt-rearing unit, age at first estrus (210 ± 0.3) was registered. When moving the gilts to the breeding unit, date, gilt weight, and P2-backfat was registered. At insemination, date and P2 backfat were registered. Throughout gestation, culled and repeating gilts were registered in order to calculate the farrowing rate, and percentage of gilts repeating. In the farrowing unit, date of farrowing, number of total born, live-born, and stillborn piglets per litter were registered.

### Statistical Analyses

All statistical analyses were performed in SAS (SAS Enterprise Guide 7.1, SAS Inc, Cary, NC) with gilt as the experimental unit. For the analysis of the effect of the treatment (feeding strategy prior to the first service) on the normally distributed production parameters, age at first detected estrus, age, weight, and backfat thickness when entering the breeding unit, days from entry to start of altrenogest, age at the initiation of altrenogest treatment, backfat thickness termination of altrenogest treatment, days from altrenogest withdrawal to insemination, and average age at first insemination, the MIXED procedure was used with the following linear mixed model:

$$\begin{array}{l} Y_{ij}=\mu +\alpha_{i}+A_{j}+e_{ij} \end{array}$$

where $Y_{ij}$ is the response variable, μ is the intercept, $\alpha_{i}$ is the fixed effect of feeding strategy prior to first service (*i* = LL, HH, HL, and LH), $A_{j}$ is the random effect of week of first estrus detection, and $e_{ij}$ is the residual error.

The analysis of the effect of the feeding strategy prior to the first service on litter size (total born piglets) was carried out using the MIXED procedure assuming normal distribution and the following model:

$$\begin{array}{l} Y_{ijk}=\mu +\alpha_{i}+\beta_{j}+\alpha {\left(\tau \;*\;x\right)}_{i}+\gamma \;*\;z_{i}+A_{k}+e_{ijk} \end{array}$$

where $Y_{ijk}$ is the response variable, μ is the intercept, $\alpha_{i}$ is the fixed effect of feeding strategy prior to first service (*i* = LL, HH, HL, and LH), $\beta_{j}$ is the fixed effect of breeding origin (*j* = before and after the change of genetics), τ and γ are the regression coefficients, $x_{i}$ is the gilt backfat when entered in the insemination section, $z_{i}$ is the covariate of gilt weight when entered in the insemination section, $A_{k}$ is the random effect of week of first estrus detection, and $e_{ijk}$ is the residual error.

The analysis of the effect of the feeding strategy prior to the first service on live-born piglets per litter, length of the gestation period, and age at first farrowing were analyzed using the MIXED procedure assuming a normal distribution using the following model:

$$\begin{array}{l} Y_{ijk}=\mu +\alpha_{i}+\beta_{j}+A_{k}+e_{ijk} \end{array}$$

where $Y_{ijk}$ is the response variable, μ is the intercept, $\alpha_{i}$ is the fixed effect of feeding strategy prior to first service (*i* = LL, HH, HL, and LH), $\beta_{j}$ is the fixed effect of breeding origin (*j* = before and after the change of genetics), $A_{k}$ is the random effect of week of first estrus detection, and $e_{ijk}$ is the residual error.

The analysis of the effect of the feeding strategy prior to the first service on stillborn piglets per litter were analyzed using the GLIMMIX procedure assuming a Poisson distribution using the following model:

$$\begin{array}{l} Y_{ijk}=\mu +\alpha_{i}+\beta_{j}+A_{k}+e_{ijk} \end{array}$$

where $Y_{ijk}$ is the response variable, μ is the intercept, $\alpha_{i}$ is the fixed effect of feeding strategy prior to the first service (*i* = LL, HH, HL, and LH), $\beta_{j}$ is the fixed effect of breeding origin (*j* = before and after the change of genetics), $A_{k}$ is the random effect of week of the first estrus detection, and $e_{ijk}$ is the residual error.

The effect of treatment (feeding strategy prior to the first service) on the binary parameters; gilts that were reinseminated, culling rate, and farrowing rate and the binomial distributed stillborn as a percentage from total born piglets were analyzed by the GLIMMIX procedure assuming a binomial distribution. The following logistic model was used to estimate the effect of feeding strategy prior to the first service:

$$\begin{array}{l} logit\left(Y_{ijk}\right)=\alpha_{i}+\beta_{j}+A_{k}+e_{ijk} \end{array}$$

where $Y_{ijk}$ is the response variable, $\alpha_{i}$ is the fixed effect of feeding strategy prior to first service (*i* = LL, HH, HL, and LH), $\beta_{j}$ is the fixed effect of breeding origin (*j* = before and after the change of genetics), $A_{k}$ is the random effect of week of first estrus detection, and $e_{ijk}$ is the residual error.

All pairwise comparisons between feeding strategies prior to the first service were adjusted with the Tukey–Kramer correction. For all data, results were considered statistically significant when *P* < 0.05 and as trends when 0.05 < *P* ≤ 0.10. Values above *P* > 0.10 were reported as nonsignificant. The results were reported as the least square means (LSMeans) with SEMs.

## RESULTS

### Results From the Breeding Unit

After the altrenogest treatment, 69.1% of the gilts were inseminated within 0–7 d and 88.8% (2101 gilts) were inseminated within 0–10 d, and the remaining 11.2% were excluded from the data set. In total, 264 gilts were excluded, and the distribution was 68 (11.3%), 64 (10.6%), 68 (12.0%), and 64 (10.8%) in group LL, HH, HL, and LH, respectively. Table 3 shows the characteristics of the gilts at insemination in all treatments. There was no difference on the age, weight, or backfat of the gilts when moved to the insemination section (age; *P* = 0.271, weight; *P* = 0.553, backfat; *P* = 0.121). The average gilt age when starting the altrenogest treatment did not differ between the groups (*P* = 0.279). The majority of the gilts were inseminated at days 241–260 and the average age at first insemination did not differ between treatments (*P* = 0.314). The time from withdrawal of altrenogest and until the first insemination was higher for gilts in the LL group than HH gilts (*P* < 0.016; Table 3). The two groups (HH and HL) that had the longest periods with a high feed allowance (on average 23–25 d for HH and precisely 18 d for HL) and thereby had an increased number of days with an increased feed allowance, they also had an increased backfat depth after altrenogest treatment (*P* < 0.001) than the gilts in group LL and LH.

**Table 3.** Characteristics of gilts at entry into the breeding unit and results at insemination for all treatment groups (low–low energy levels, LL; high–high energy levels, HH; high–low energy levels, HL; and low–high energy levels, LH) for gilts that were inseminated 0–10 d after altrenogest withdrawal^*a*^

	Group					
	LL	HH	HL	LH	SEM	*P*-value
First identified estrus						
Age, days	210	211	211	210	2.02	0.312
Gilts, no.^*b*^	533	536	500	528		
Entry in the breeding unit						
Age, days	215	216	216	215	2.01	0.271
Weight, kg	148.8	148.4	149.3	149.5	1.48	0.553
Backfat (P2), mm	15.3	15.2	15.1	14.9	0.23	0.121
Treatment with altrenogest						
Time from entry to start of altrenogest, days	4.9	4.9	4.9	4.9	0.01	0.933
Age at initiation, days	220	221	221	220	2.02	0.279
Backfat (P2) at termination of treatment, mm	15.7^a^	16.2^b^	16.2^b^	15.8^a^	0.20	<0.001
Insemination						
Time from altrenogest withdrawal to insemination, days	6.89^a^	6.68^b^	6.75^ab^	6.75^ab^	0.08	0.016
Average age, days	244	244	245	244	1.99	0.314
Gilts, no.^*c*^	533	536	500	528		

^*a*^Results are presented as LSMeans ± SEM.

^*b*^Gilts excluded from data when entering the breeding unit were due to the following reasons: less than 9 mm of backfat, locomotive problems, abnormal discharge, or coughing.

^*c*^Gilts were excluded from data when days from altrenogest withdrawal to insemination exceeded 10 d and accounted for 68, 64, 68, and 64 gilts in groups LL, HH, HL, and LH, respectively.

^a,b^Values within a row lacking a common superscript letter differ (*P* < 0.05; Tukey–Kramer adjusted).

### Production Results

No differences were found in the reproduction results between groups (Table 4). On average, only 0.5% of the gilts were reinseminated and approximately 4.6% of the gilts were culled as nonpregnant between inseminations and farrowing due to either abnormal discharge or coughing. The farrowing rate based on the first insemination was 94.0–95.6% for the gilts and did not differ between treatments (*P* = 0.647; Table 4). The length of the gestation period was likewise unaffected by treatment (*P* = 0.610).

**Table 4.** Gilt performance results after the use of altrenogest and different feeding strategies for all treatment groups (low–low energy levels, LL; high–high energy levels, HH; high–low energy levels, HL; and low–high energy levels, LH) for gilts that were inseminated 0–10 d after altrenogest withdrawal^*a*^

	Group					
	LL	HH	HL	LH	SEM	*P*-value
Gestation						
Culled after insemination, %^*b*,*c*^	4.6 [3.1;6.9]	4.4 [2.8;6.6]	3.4 [2.1;5.5]	5.0 [3.4;7.4]	–	0.600
Repeating gilts, %^*b*^	0.4 [0.09;1.5]	0.6 [0.2;1.7]	0.4 [0.1;1.6]	0.6 [0.2;1.8]	–	0.951
Farrowing rate, %^*b*^	95.0 [92.6;96.6]	94.3 [91.9;96.1]	95.6 [93.3;97.2]	94.0 [91.4;95.8]	–	0.647
Gestation period, days	117	117	117	118	0.08	0.610
Farrowing						
Farrowings, no.	506	507	478	500	–	
Total pigs born/litter, no.	16.0	16.1	16.0	16.4	0.16	0.069
Live-born/litter, no.	15.6	15.7	15.7	16.0	0.16	0.229
Stillborn pigs/litter, no.^*b*^	0.3 [0.28;0.39]	0.4 [0.33;0.45]	0.3 [0.27;0.38]	0.4 [0.31;0.43]	–	0.259
Stillborn pigs of total born, %^*b*^	2.1 [1.7;2.6]	2.4 [2.0;2.9]	2.0 [1.6;2.5]	2.3 [1.9;2.8]	–	0.509
Age at farrowing, days	362	362	362	362	1.97	0.525

^*a*^Results are presented as LSMeans ±SEM.

^*b*^The deviations of nonnormally distributed variables are given as 95% confidence interval.

^*c*^Gilts hat were culled after insemination were all nonpregnant and culled due to either abortion and/or abnormal discharge or coughing.

^a,b^Values within a row lacking a common superscript letter differ (*P* < 0.05; Tukey–Kramer adjusted).

**Figure 1.**
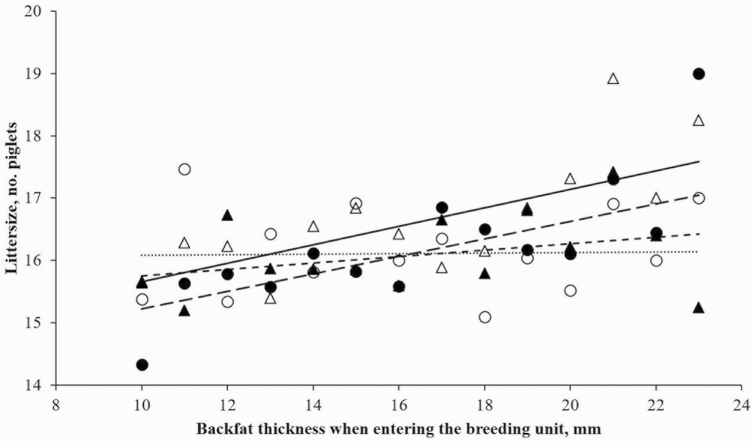
The effect of feeding strategy and backfat thickness when entering the breeding unit on littersize for all treatment groups. The dots and squares indicate the mean littersize for all sows within each backfat depth and treatment group. The lines indicate the prediction regression for all treatment groups [low–low energy levels, LL *●* and solid line (――); high–high energy levels, HH *○* and dotted line (∙∙∙∙∙); high–low energy levels, HL *▲* and dashed line (- - -); and low–high energy levels, LH *∆* and long-dashed line (― ― ―)].

**Figure 2.**
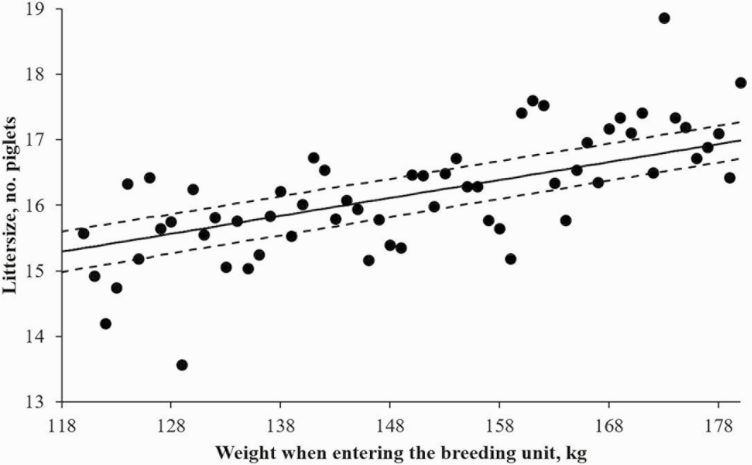
The effect of weight when entering the breeding unit on littersize for all treatment groups. Each dot (*●* ) indicates the mean littersize for all sows within a given weight. The solid line indicates the prediction regression line and the dotted lines indicate the standard error.

Increasing the feeding level only in the follicular phase (LH) tended to increase a higher number of total born piglets per litter (0.3–0.4 total born piglets per litter) compared to the other groups (*P* = 0.069; Table 4). A tendency to an interaction between feeding strategy prior to the first service and backfat at the entry to the breeding unit was observed (*P* = 0.076; Fig. 1) showing that total born piglets per litter tended to be positively correlated to backfat at entry in the LL and LH groups (no increase in feed allowance or a short-term increase in the feed allowance prior to the first service), whereas BF had less influence on total born piglets per litter when sows had longer periods with increased feed allowance prior to first service (HH and HL groups). The weight of the gilt when entering the insemination section also had an effect on total born piglets (*P* < 0.001) with an increase in litter size with increased weight of the sow (Fig. 2) but no differences between treatments. The treatment had no effect on either liveborn piglets per litter (*P* = 0.229) or stillborn piglets per litter (*P* = 0.259) or stillborn expressed as the percentage of total born piglets (*P* = 0.509).

## DISCUSSION

Increasing feed allowance prior to insemination secures a sufficient release of follicles at insemination. The primary effect of the increased feed allowance is the increase of insulin (Beltranena et al., 1991b; Almeida et al., 2001; Almeida et al., 2014) that then stimulates the production of follicular hormone and luteinizing hormone (LH). Thus, insulin has an indirect stimulating effect on both the number and size of follicles ovulated (Beltranena et al., 1991a; Almeida et al., 2014). Increasing the number of follicles ovulated increases the potential of more liveborn (Close and Cole, 2000). Previously, studies investigating energy levels on flushing (Schultz et al., 1966; Flowers et al., 1989; Beltranena et al., 1991a, 1991b), timing of flushing in the gilts cycle (Grandhi et al., 1987; Flowers et al., 1989; Beltranena et al., 1991a; Hazeleger et al., 2005), or very increased feed levels (Grandhi et al., 1987; Beltranena et al., 1991b) have been conducted. However, despite these studies, it is still not clear when the feed allowance should be increased to maximize ovulation rate in gilts inseminated in their second heat, and, furthermore, the effects may be affected by the use of hyper prolific genetics and also by the use of altrenogest to synchronize gilts prior to the first service in the second estrus.

The aim of this study was to investigate different feeding strategies covering different time points of the estrus cycle in order to optimize the time frame of increased feed allowance prior to the first service in gilts when using altrenogest to control the luteal phase and initiation of the follicular phase. In order to get the maximum out of a high feed allowance, it should be terminated when the gilts have been inseminated. One of the reasons for this is the loss of embryos if the high feeding level is continued (Jindal et al., 1996; Langendijk, 2015).

The LH gilts tended to have the highest litter size. Providing a high feed allowance just before insemination has previously been shown to increase litter size (Close and Cole, 2000; Almeida et al., 2014; Knox, 2019). In addition, a high feed allowance in the follicular phase (equivalent to the last 5–7 d before insemination in the second estrus) of the gilts cycle has the biggest impact on the amount of ovulated follicles (Dailey et al., 1975) in accordance with the current study. This is believed to be due to a higher secretion of insulin when increasing the feed allowance and is further supported by Almeida et al. (2014), who showed that flushing with corn starch from day 8 in cycles and until next estrus (covering parts of the luteal phase and the entire follicular phase) resulted in elevated levels of insulin on days 14 and 21 and increase in the ovulation rate of 2.6 follicles (+18.8%) and embryo numbers (+17.5%), as well as embryo weight (+17.6%), compared with flushing in the same period using soybean oil. This clearly shows that insulin is a major driver of follicle development and growth. However, based on the findings of Chen et al. (2012), it would have been expected to find a higher ovulation rate in gilts fed the HH in our study because increasing the feeding level by 1.5 kg/d was shown to increase embryo number at days 9–9.5 after the third ovulation in gilts (Chen et al., 2012). The reason for not finding this effect is most likely because the gilts in the current study given a low feed allowance were still fed well above maintenance (2.33 kg/d), whereas the low feeding level used by Chen et al. (2012) was around 1.2 kg/d. Feeding gilts close to maintenance in any part of their cycle prior to the first insemination should therefore be avoided.

The higher backfat depth in HH and HL compared to LH and LL gilts could influence litter size. Currently, gilts are inseminated with a lower backfat depth than previously, which could result in a more powerful response to the feeding strategy prior to the first service than earlier, as studies have shown that gilts that were in a good body condition responded less positively to a high feed allowance than thin animals (Beltranena et al., 1991a). The gilts in the present study had a relatively high backfat depth at insemination (15.7–16.2 mm) compared to a recent Danish study (Klaaborg et al., 2019) and it can therefore be speculated that the response at a lower backfat depth would have been even greater. The litter size of the HL group (high feed allowance in the luteal phase) indicates that even though the gilts were given additional feed in the prefollicular phase then the lack of a high feed allowance in the follicular phase contributes to no extra follicles being ovulated or that the quality was degraded so that there were not fertilized more follicles than in the control group (LL). As stated above, this is most likely due to the low feeding level being well above maintenance.

Although the gilts were not weighed at insemination in order not to disturb implantation, the current study shows that the gilts that were of a higher weight when entering the breeding unit had more total piglets born per litter. Recently, a Danish study with 1406 DanBred gilts found that for each 10 kg increase in weight at first insemination, litter size was increased by 0.4 total born piglets per litter. Further analyses revealed that neither age at first service nor backfat thickness or any interactions with weight when entering the breeding unit significantly affected litter size (Bruun et al., 2020).

One of the main challenges in implementing feeding strategies prior to the first service is the variation in time of gilts showing standing response within a pen and thereby insemination times. Altrenogest synchronizes the beginning of the follicular phase and, therefore, the different feeding strategies could be determined more precisely as the gilts were in the luteal phase as long as they were receiving altrenogest and first entered the follicular phase when the altrenogest treatment stopped. In addition, the length of altrenogest treatment was 18 days for all gilts, thereby making sure that they were as synchronized as possible (Stevenson and Davis, 1982). However, although using altrenogest reduced this variation in time of showing standing response, there was still a variation in days between gilts of 4–10 d. By using altrenogest to suppress estrus or as in the current study to synchronize estrus, it is expected that 80% of the gilts will come into estrus 4–8 d after finished treatment (Martinat-Botté et al., 1995; Soede et al., 2007; Ziecik et al., 2017). The use of altrenogest caused 88.8% of the gilts to be inseminated within 10 d after withdrawal. In agreement, Wood et al. (1992) found that 86% of the gilts that were treated with altrenogest came into estrus 0–7 d after end treatment and 92% when including up to day 10 (Wood et al., 1992). The time of initiating the altrenogest treatment in the gilt cycle may affect the number of days from withdrawal to the first insemination. Stevenson and Davis (1982) showed that starting the altrenogest treatment from 1 d before to 2 d after estrus increased the time from the end of treatment to estrus by 0.4 d compared with treatments started during the diestrus period (days 3–21), though this slight delay in estrus 97–100% in both groups were serviced within 10 d irrespective of whether the treatment period was 14 or 18 d (Stevenson and Davis, 1982). With this in mind, the increased time from withdrawal of altrenogest to the first insemination in the LL group compared with the HH group (0.2 days) is not particularly interesting as this will not influence any results. It is though surprising that when all gilts were cyclic before the use of altrenogest, the efficiency in terms of gilts serviced within 10 d after the treatment were well below the potential 97–100%.

The number of stillborn piglets per litter (2.1–2.4% of total born per litter) was at a very low level compared to other studies carried out using the same genetics first-parity sows (Klaaborg et al., 2019). This could be due to the 24-h farrowing surveillance on the farm. Furthermore, sows were adopted to environment and feed changes for 5 d prior to the expected farrowing date, which might be beneficial for farrowing performance. Furthermore, an optimum body condition score in terms of backfat was in focus on the current farm, and, in general, too lean sows at farrowing were avoided, which may have contributed to less stillborn piglets being born (Maes et al., 2004).

By increasing the daily feed allowance, the gilts will receive a substantial increase in the daily energy intake—for example, more than 20% extra feed, and preferably even more, although ensuring that the gilts can eat the given amount. In the current trial, feed allowance was increased by 42% in the periods with a high feed allowance, and because of a calculated content of starch and sugars around 46.7% in the diet (data not shown), this should contribute to an increase in blood glucose and thus insulin response compared to the LL group, which in terms is supported by the data from Almeida et al. (2014) and might, therefore, be an explanation to the results. Other studies have found differences in plasma insulin and glucose concentrations depending on feeding levels; for example, Quesnel et al. (1998) and Koketsu et al. (1996) found differences when the low feeding level was approx. 50% of the feeding level. In contrast, van den Brand et al. (2000) did not find differences between insulin and glucose concentrations, but feeding levels only differed by 25%. This indicates that feed allowance should be increased drastically in order to induce the flushing effect; furthermore, the diet should be rich in starch, which is further supported by Kemp et al. (1995), who found that in starch-fed sows compared to fat-fed sows, luteinizing hormone pulsatility at day 7 of lactation was greater (*P* < 0.05), the preovulatory luteinizing hormone surge was greater (*P* < 0.05), and progesterone production was greater (*P* < 0.05) from 108 h until 256 h after the luteinizing hormone surge in the starch-fed sows. This reveals that the early follicular development may be better supported when the feeding strategy aims to increase insulin secretion and thereby affect the luteinizing hormone.

From this current study, there are some optimal gilt management strategies worth discussing. Reducing the pen variation of when gilts show standing response is a key management tool. This is to avoid that there are gilts in the pen needing a high feed allowance together with gilts that have already been inseminated and therefore need reduced feed levels in order to reduce embryo loss (Einarsson et al., 1996). Altrenogest was used to synchronize the gilts in the current study; however, the variation in days from the finished altrenogest treatment to insemination suggests that there will be newly inseminated gilts in the pens when the last ones react to the altrenogest treatment. In addition, they were housed individually, allowing individual feed adjustments, although this would not be possible as a common practice within the EU due to legislation.

Providing the gilts with a high feed allowance for the last 10–13 d before expected insemination could be an alternative to ensure that close to all gilts experience a high feeding level for at least the last 5–7 d prior to expected insemination, and this will furthermore contribute to some degree of flushing in the prefollicular phase, which could be beneficial especially for thinner gilts.

In conclusion, we found a tendency toward a positive correlation between backfat in the LL and LH groups (no increased feed allowance or an increased feed allowance in the follicular phase) on total born piglets per litter, whereas BF had less influence on total born piglets per litter when sows had a longer period with increased feed allowance (HH and HL groups). In addition, a high feed allowance in the follicular phase (LH) tended to result in a higher number of total born piglets per litter (0.4 total born piglets per litter) compared to the other treatments. This would be equivalent to the last 5–7 d of a 21-d cycle in gilts. Finally, the weight of the gilt when entering the breeding unit also increased litter size. These results suggest that management strategies prior to the first service in the second estrus should take the weight of the gilt and feed allowance into account as different strategies might be warranted depending on the body condition of the gilt.
